# Inhibition of the Quorum Sensing System (ComDE Pathway) by Aromatic 1,3-di-m-tolylurea (DMTU): Cariostatic Effect with Fluoride in Wistar Rats

**DOI:** 10.3389/fcimb.2017.00313

**Published:** 2017-07-12

**Authors:** Gurmeet Kaur, P. Balamurugan, S. Adline Princy

**Affiliations:** Quorum Sensing Laboratory, Centre for Research in Infectious Diseases, School of Chemical and Biotechnology, SASTRA University Thanjavur, India

**Keywords:** quorum sensing, dental caries, antibiofilm, multi-drug resistance, DMTU

## Abstract

Dental caries occurs as a result of dysbiosis among commensal and pathogenic bacteria leading to demineralization of enamel within a dental biofilm (plaque) as a consequence of lower pH in the oral cavity. In our previous study, we have reported 1,3-disubstituted ureas particularly, 1,3-di-m-tolylurea (DMTU) could inhibit the biofilm formation along with lower concentrations of fluoride (31.25 ppm) without affecting bacterial growth. In the present study, RT-qPCR analysis showed the target specific molecular mechanism of DMTU. *In vivo* treatment with DMTU, alone or in combination with fluoride, resulted in inhibition of caries (biofilm development of *Streptococcus mutans*) using a Wistar rat model for dental caries. The histopathological analysis reported the development of lesions on dentine in infected subjects whereas the dentines of treated rodents were found to be intact and healthy. Reduction in inflammatory markers in rodents' blood and liver samples was observed when treated with DMTU. Collectively, data speculate that DMTU is an effective anti-biofilm and anti-inflammatory agent, which may improve the cariostatic properties of fluoride.

## Introduction

*Streptococcus mutans* is known as one of the principal aetiological agent that plays a significant role in the transition of non-pathogenic commensal oral microbiota to highly acidic and cariogenic biofilms resulting in the development of dental caries. Worldwide, dental caries is one of the most common biofilm-dependent oral infectious diseases. The major virulence factors include acidogenicity and aciduricity along with its characteristic ability to produce dental plaque (biofilm). In *S. mutans*, biofilm formation is regulated by quorum sensing (QS) that involves ComDE two-component signal transduction system (TCSTS) which regulates the expression of virulence factors in cell density dependent manner. ComDE QS circuit in *S. mutans* specifically responds to the competence stimulating peptide (CSP; Kaur et al., 2015). The CSP is synthesized as a 21 amino acids propeptide by *comC* followed by maturation of CSP by an ABC transporter ComA along with an accessory protein ComB and finally secreted (18 amino acids long peptide signal) to the extracellular environment. Secreted peptide is detected by the histidine kinase membrane-bound protein receptor, ComD, resulting in phosphorylation of its cytoplasmic response regulator, ComE thus, resulting in expression of various virulence genes as a response to signaling peptide (Ishii et al., 2010).

Biofilm formation protects bacteria from the host immune system and also acts a diffusion barrier providing resistance to bacteria from various antimicrobials (Senadheera and Cvitkovitch, 2008; Arya and Princy, 2013). In fact, cells existing within the biofilm community are 10–1,000 times more resistant to antimicrobials than their planktonic counterparts (Mah and O'Toole, 2001). Dental plaque (biofilm), if allowed to persist on tooth surfaces, subsequently progress to the development of periodontitis leading to the extraction of a tooth in infected individuals. *S. mutans* may enter the blood stream via injuries in oral cavity and further attach to platelet-fibrin-matrices on damaged endothelial tissue. The ability of *S. mutans* to adhere and thrive on the injured heart tissue leads to the unhindered survival and pathogenesis of chronic infective endocarditis which may cause significant morbidity and mortality (Bansal et al., 2013). Invasion by *S. mutans* in the blood stream may also result in bacteremia causing chronic inflammation and its manifestations such as rheumatoid arthritis, premature birth of babies. Targeting one of the key components involved in cell-cell signaling process can lead to inhibition of biofilm formation (Qi et al., 2005; Rasmussen and Givskov, 2006; Ravichandiran et al., 2012). Development of novel anti-biofilm drugs against biofilm forming bacteria without causing mortality of the pathogen might result in the inhibition of biofilm formation (Balamurugan et al., 2015; Chen et al., 2015). In this context, we have previously reported ComA as a potential target for drug development (Kaur et al., 2016). *In silico* findings showed 1,3-disubstituted ureas as potential ligands followed by synthesis and *in vitro* validation of parent ligand (DMTU) along with five derivative ligands revealed parent ligand i.e., 1,3-di-m-tolylurea (DMTU) as a potential inhibitor of ComA. Fluoride has been used long as an effective cariostatic agent for caries prevention in commercial formulations. However, prolonged use of high concentrations of fluoride (1,000–2,000 ppm) has led to the development of fluoride-resistant strains along with its reported side effects such as fluorosis, neurotoxicity, and weakened bones in children (Jetti et al., 2014; Spittle, 2016). Therefore, to explore the possibility of reducing the fluoride concentration, we had included fluoride in our *in vitro* assays and investigated its synergistic activity along with our synthesized compounds. Results of *in vitro* validation indicated that DMTU could act as a potent biofilm inhibitor alone as well as along with lower concentration of fluoride (31.25 ppm) among all the synthesized compounds.

Thus, to further explore the target specific activity of DMTU, the objectives of the present study were (i) to elucidate the target specific mechanism of DMTU on various quorum regulated genes and (ii) to test and validate the activity of DMTU at pre-clinical stages using Wistar rat model for dental caries.

## Materials and methods

### Cell culture

Liver hepatocellular carcinoma (Hep G2) cells were received from National Centre for Cell Science (NCCS), Pune, India. The cells were cultured in Dulbecco's modified Eagle's medium (HiMedia) supplemented with 10% fetal bovine serum (FBS) and 1% pen/strep. The cells were incubated and maintained at 37°C in a saturated humidified incubator with 5% CO_2._ MTT [3-(4,5-dimethylthiazole-2-yl)-2,5-diphenyl tetrazolium bromide] for cell proliferation assay was purchased from HiMedia, Mumbai, India.

### Bacterial strains and growth medium

*S. mutans* MTCC 497 was received from the Microbial Type Culture Collection (MTCC), Chandigarh, India and was used as a standard strain in the study. A clinical isolate of *S. mutans*, SM4 (Multidrug resistant) was received from JSS Medical College, Mysore, India. The SM4 strain was categorized to be multidrug resistant as described by Magiorakos et al. (2012). Both the strains were grown at 37°C in brain heart infusion broth/agar (HiMedia) supplemented with 2% sucrose.

### Test compounds

**Figure 1:**
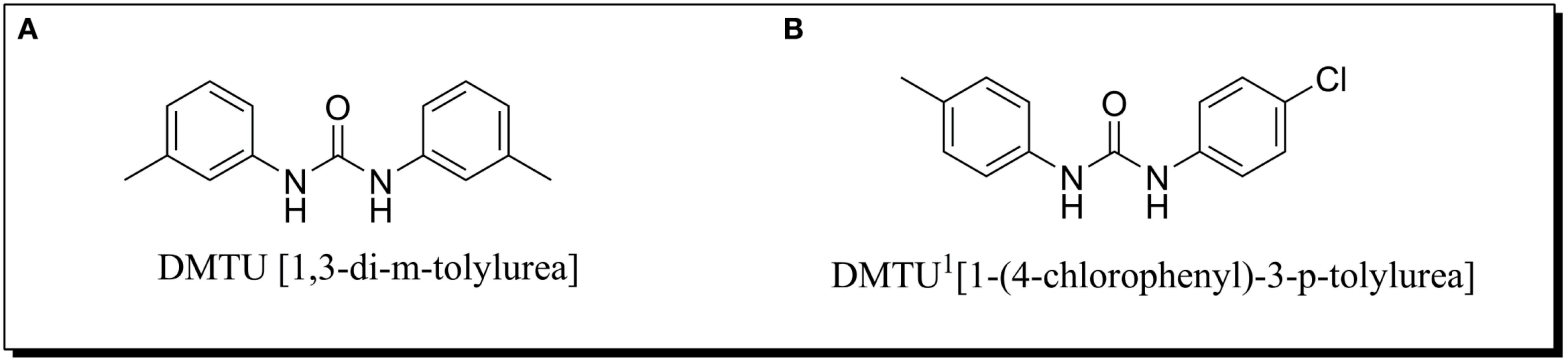
Chemical structure of **(A)** DMTU (1,3-di-m-tolylurea) and **(B)** DMTU^1^ (1-(4-chlorophenyl)-3-p-tolylurea).

The synthesis of aromatic 1,3-disubstituted ureas was carried out by a simple one-pot reaction of aryl isocyanates with the selective amines has been reported previously by the authors. Further, the derivatives of the lead compound were synthesized and screened *in vitro* for their anti-biofilm activity. Amongst all the synthesized compounds, DMTU (1,3-di-m-tolylurea; Figure 1A) was found to be the most effective based on their biofilm inhibiting activity against *S. mutans*.

### Quantification of gene expression using RT-qPCR

*S. mutans* (MTCC 497 and SM4) were grown in BHIB in the presence and absence of DMTU (3.75 μM) and fluoride (31.35 ppm) till 24 h at 37°C. Cells were harvested by centrifugation from grown cultures (0.5 ml) at early log phase (3–5 h), mid-log phase (8–10 h) and stationary phase (24 h) and immediately stored at −80°C. RNA was isolated using a Qiagen RNeasy mini Kit in accordance with the manufacturer's instructions. RNA concentrations were determined by OD_260_ measurements in a NanoDrop (Thermo Scientific, USA). cDNA synthesis was carried out using the iScript™ cDNA Synthesis Kit according to the manufacturer's instructions. Briefly, the reaction mixture was incubated for annealing at 25°C for 5 min, extension at 42°C for 30 min and inactivation of samples at 85°C for 5 min.

RT-qPCR was used to assess the transcription levels of biofilm and virulence related genes (*comA, nlmC, immA, immB, bsmH, bsmI, comDE, comX, comB*). Sequences of the primers used in this study are furnished in Table 1. The reaction mixture in a total volume of 20 μl, consisted 10 μl 2X SYBR Green PCR Master Mix, forward and reverse primers (1 μl each), 4 μl of nuclease-free water and 4 μl of 20X diluted cDNA (Hasan et al., 2012). PCR conditions included an initial denaturation at 95°C for 2 min, followed by 40 cycles of denaturation (95°C for 15 s), annealing (55–57°C for 15 s), and extension (72°C for 20 s). To ensure the samples were free from contamination, negative controls containing nuclease-free water instead of cDNA were run in parallel. The relative gene expression was analyzed using the 2^−ΔΔCT^ method with 16S r-RNA as a reference gene. RT-qPCR experiments were in compliance with the MIQE (Minimum Information for Publication of Quantitative Real-Time PCR Experiments) guidelines as listed in the MIQE checklist (Bustin et al., 2009; Supplementary Information: Table S1).

**Table 1:** Primer sequence for RT-qPCR analysis of various genes in *S. mutans*.

**Gene**	**Forward Primer Sequence (5′–3′)**	**Reverse Primer Sequence (5′–3′)**
*comDE*	ACAATTCCTTGAGTTCCATCCAAG	TGGTCTGCTGCCTGTTGC
*16S rRNA*	CCTACGGGAGGCAGCAGTAG	CAACAGAGCTTTACGATCCGAAA
*nlmC*	TTGTGCAGCAGGTATTGCTC	AAGAGCTCCTCCGATTCCTC
*immA*	TCTCCCCTGCTTGTTCAGAT	GCTGGCAAATTCGCTTACTT
*immB*	GCTAGAGAGGCAAATGCACA	CAGCAGCAGCTGAGAAGATG
*bsmI*	GAAACAATGGATACAGAGACG	GGAACAATAAGAGGATTTGG
*bsmH*	AGACATGTTAGCCGCTGTTGAAG	AAGCGCCTGTTCCAATCGTA
*comX*	CTGTTTGTCAAGTGGCGGTA	GCATACTTTGCCTTCCCAAA
*comA*	ACGAGCCTAACAAGGGGATT	CCCTGAGGCATTTGTTCAAT
*comB*	CCAGTCCAAACCGTCAGACT	GCTGCTTTCCTTGTCTTTCG

### MTT cell proliferation assay

For toxicity analysis, Liver hepatocellular carcinoma (Hep G2) cells were cultured in the presence of DMTU and DMTU^1^ Figure 1B. After trypsinization and counting the cells with a hemocytometer, 10,000 cells were seeded in 96 well plate along with 1, 3-disubstituted ureas. The cells were allowed to proliferate for 24 h at 37°C and at the endpoint, MTT reagent was added and further subjected to 4 h of incubation (Ciofani et al., 2010). The formazan crystals formed after addition of MTT were solubilized using DMSO (100 μL) and the absorbance was measured at 570 nm in a microtitre plate reader (iMark, BIORAD, Japan).

### Animal study

#### Acute oral toxicity (AOT) analysis:

Healthy female Wistar rats aged 8 weeks used for the AOT analysis were bred and reared in the Central Animal Facility, SASTRA University, Thanjavur, Tamil Nadu, India. The animals were acclimatized to animal house conditions for 1 week prior to the treatment with DMTU. The animals were housed and maintained in polypropylene cages consisting of clean paddy husk bedding with stainless steel grill lids at a temperature of 25 ± 2°C under a 12:12 h light-dark cycle. The rats were fed with pelleted feed (M/S ATNT Laboratories, Mumbai, India) and filtered tap water *ad libitum* throughout the experiment.

The acute oral toxicity test of DMTU was evaluated in rats using the up and down procedure in accordance with OECD 425 guidelines (Maneewattanapinyo et al., 2011). Briefly, the rats were divided into five groups with the first group receiving a limited dose of 175 mg/kg orally using a suitable intubation canula. The animals were observed for toxic symptoms continuously for the first 3 h after dosing. The animals were further observed for 48 h and based on survival of the first group rats, the second group was dosed with 550 mg/kg orally. Similar observations were carried out for the second group and subsequently based on the survival of rat, the dosing was increased to 2,000 mg/kg for the next three groups. All these animals were then maintained for 14 days further with feed intake observations made on a daily basis and weight observations on a weekly basis. At the 14th day, the animal was sacrificed and vital organs were observed macroscopically by a calibrated professional histopathologist for any lesions.

#### Efficacy studies

The animal experiments were reviewed and approved by Institutional Animal Ethics Committee (IAEC) with approval number 382/SASTRA/IAEC/RPP of SASTRA University, Thanjavur, Tamil Nadu, India and was performed according to the methods described previously (Murata et al., 2010). To determine the effects of DMTU on caries establishment, a total of 42 SPF female Wistar rats aged 21 days were purchased from the Central Animal Facility, SASTRA University, Thanjavur, India. After acclimatization for 5 days, the 30 animals were infected with clinical isolate of *S. mutans* SM4, using a sterile cotton swab dipped in culture medium (10^5^ CFU/mL) and randomly divided into five groups (*n* = 6 per group): a disease control, a DMTU treated group (3.75 μM), a fluoride treated group (500 ppm), a synergy group (3.75 μM DMTU and 31.25 ppm fluoride), a 10 X DMTU group (37.5 μM- to determine the long-term effects of high dose of DMTU). The swab was obtained and plated on Mitis Salivarius Agar with 0.2 U/mL bacitracin to confirm the colonization of *S. mutans* on dentine. Each group was fed with diet 2,000 (contains 56% sucrose) and 5% sucrose water *ad libitum*. In addition to these five groups, two other groups were maintained as controls (*n* = 6 per group): a control group without sucrose diet and another control group with diet 2,000 and 5% sucrose water. From this point, the molars of animals were given topical treatments with their corresponding concentrations once daily by using a camel hair brush. The animals were noted for their body weight weekly and physical appearance was noted daily. The treatment was carried out for 7 weeks, and at the end of the experimental period, animals were euthanized by CO_2_ asphyxiation. The lower jawline was dissected aseptically and suspended in 10 ml of sterile phosphate buffer saline and subjected to sonication (20 s pulses at 10 s intervals for two times) to recover the maximum adhered viable counts. The solution was further serially diluted and plated on Mitis Salivarius Agar with 0.2 U/mL bacitracin to estimate the *S. mutans* population. The determination of the severity of caries developed on molars of the animals was scored according to Larson's modification of the Keyes system (Larson, 1981) and was performed by expert examiner in caries evaluation.

#### Histopathological evaluation

For histopathological evaluation, the liver tissues and decalcified dentine was collected and post-fixed in 4% PFA for 24 h at 4°C, embedded in paraffin (Leica EG1150H, Leica Microsystems, Heerbrugg, Switzerland), and sectioned into ~3 μm thick sections (Leica RM2125 RTS, Leica Microsystems, Heerbrugg, Switzerland). The sections were further stained with hematoxylin and eosin using an automated tissue processing and staining system (Leica TP 1020; Leica FG1150; Leica RM 2125 RTS and Leica ST4040) and scored blindly by a veterinary pathologist to be examined under a binocular microscope (Nikon Eclipse Ci-Ds-Fi2; Cardiff et al., 2014).

#### Inflammatory parameters evaluation

Inflammatory markers were assessed using RT-qPCR method. Blood samples (5 ml each) from all the rats were collected in EDTA-treated collection tubes, just before the necropsy was performed. The blood samples were further centrifuged at 2000 rpm for 10 min at 4°C for plasma collection (Chavali et al., 2014). The plasma samples were immediately stored at −20°C until used. RNA was isolated using a Qiagen RNeasy mini kit were assessed and cDNA synthesis was carried out using the same procedure as described in Section Quantification of Gene Expression Using RT-qPCR.

RT-qPCR analysis was carried out in 96 well plates (Thermofisher) using Realplex 2 (Eppendorf) to assess the transcription levels of genes related to inflammatory markers (IL-1, IL-6, C-Reactive Protein, TNF-α; Table 2). Reaction mixture in a total volume of 20 μl, consisted 10 μl 2X SYBR Green PCR Master Mix, forward and reverse primers (1 μl each), 4 μl of nuclease-free water and 4 μl of 20X diluted cDNA. PCR conditions included an initial denaturation at 95°C for 2 min, followed by 40 cycles of denaturation (95°C for 15 s), annealing (52.9°C for 15 s), extension (72°C for 20 s). To ensure the samples were free from contamination, negative controls containing nuclease-free water instead of cDNA were run in parallel. The relative gene expression was analyzed using the 2^−ΔΔ*CT*^ method with *Gapdh* as internal control.

**Table 2:** Primer sequence used for RT-qPCR analysis of inflammatory markers in rat liver and blood.

**Gene**	**Forward Primer Sequence (5′−3′)**	**Reverse Primer Sequence (5′−3′)**
*IL1*	GGAAAGGGGAGAAATCCAAG	TGTTCTTTTCACCCCCTGAC
*IL6*	CCGGAGAGGAGACTTCACAG	ACAGTGCATCATCGCTGTTC
*CRP*	AACCTGGGAGAGGGTCAGAT	GACTCTGCTTCCAGGGACAC
*TNF-α*	AGTCCGGGCAGGTCTACTTT	GGCCACTACTTCAGCGTCTC
*Gapdh*	CATGGTCTACATGTTCCAGT	GGCTAAGCAGTTGGTGGTGC

### Statistical analysis

For RT-qPCR, one way ANOVA and multiple comparisons were performed. The data from *in vivo* study were analyzed by unpaired Student's *t*-test. For relative quantification of genes, the ΔΔCt mathematical model was used and normalization of RT-qPCR data was carried out using 16S-rRNA (for *S. mutans* genes) and *Gapdh* (for animal samples) as a reference gene by comparing the ratios of the gene of interest to those of a reference gene. The minimum level of significance was set at *p* ≤ 0.05 (95% Confidence Interval). All the assays were carried out in triplicates and the results were expressed as mean ± SD. Graph Pad Prism software (version 6.01) was used for statistical analysis for all the experiments.

## Results

### Gene expression profiling using RT-qPCR

**Figure 2:**
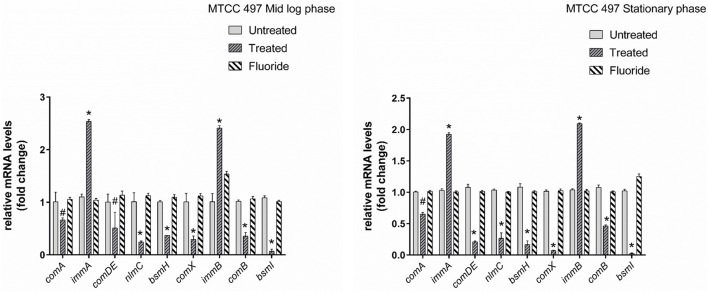
RT-qPCR analysis of various genes involved directly and indirectly in quorum sensing circuit of MTCC 497 (*S. mutans*). The results are represented as ratios corresponding to the fold change of genes treated with DMTU (3.75 μM) and fluoride (31.25 ppm) as well as control (without treatment) at mid-log phase and stationary growth phase. 16S rRNA gene was used as an internal control for data normalization. ^*^Indicates significantly different (*p* < 0.05) compared to untreated genes. #*p* > 0.05.

**Figure 3:**
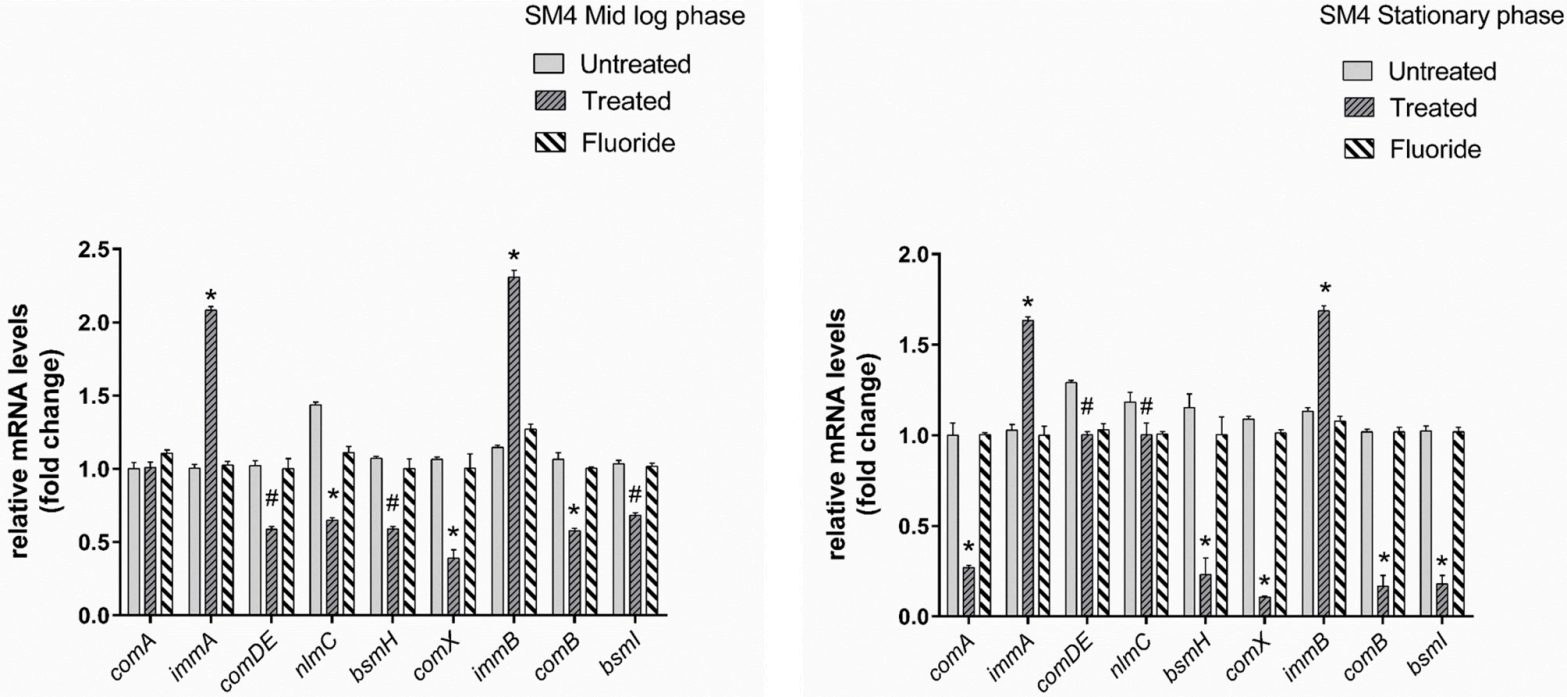
RT-qPCR analysis of various genes involved directly and indirectly in quorum sensing circuit of clinical isolate SM4. The results are represented as ratios corresponding to the fold change of genes treated with DMTU (3.75 μM) and fluoride (31.25 ppm) as well as control (without treatment) at mid log phase and stationary growth phase. 16S rRNA gene was used as an internal control for data normalization. ^*^Indicates significantly different (*p* < 0.05) compared to untreated genes. #*p* > 0.05.

Gene expression study using RT-qPCR in MTCC 497 revealed that the genes which were located downstream to *comA* were down-regulated at mid-log phase and stationary phase except for *immA* and *immB* genes which were found to be up-regulated. The genes were found to have a basal level expression at the early phase (Figure 2). In contrast, fluoride did not show any significant effect on the expression of quorum sensing genes at mid-log phase as well as the stationary phase. Similar results were achieved in SM4 strain treated with DMTU at the tested concentration (Figure 3).

### Cytotoxicity analysis

**Figure 4:**
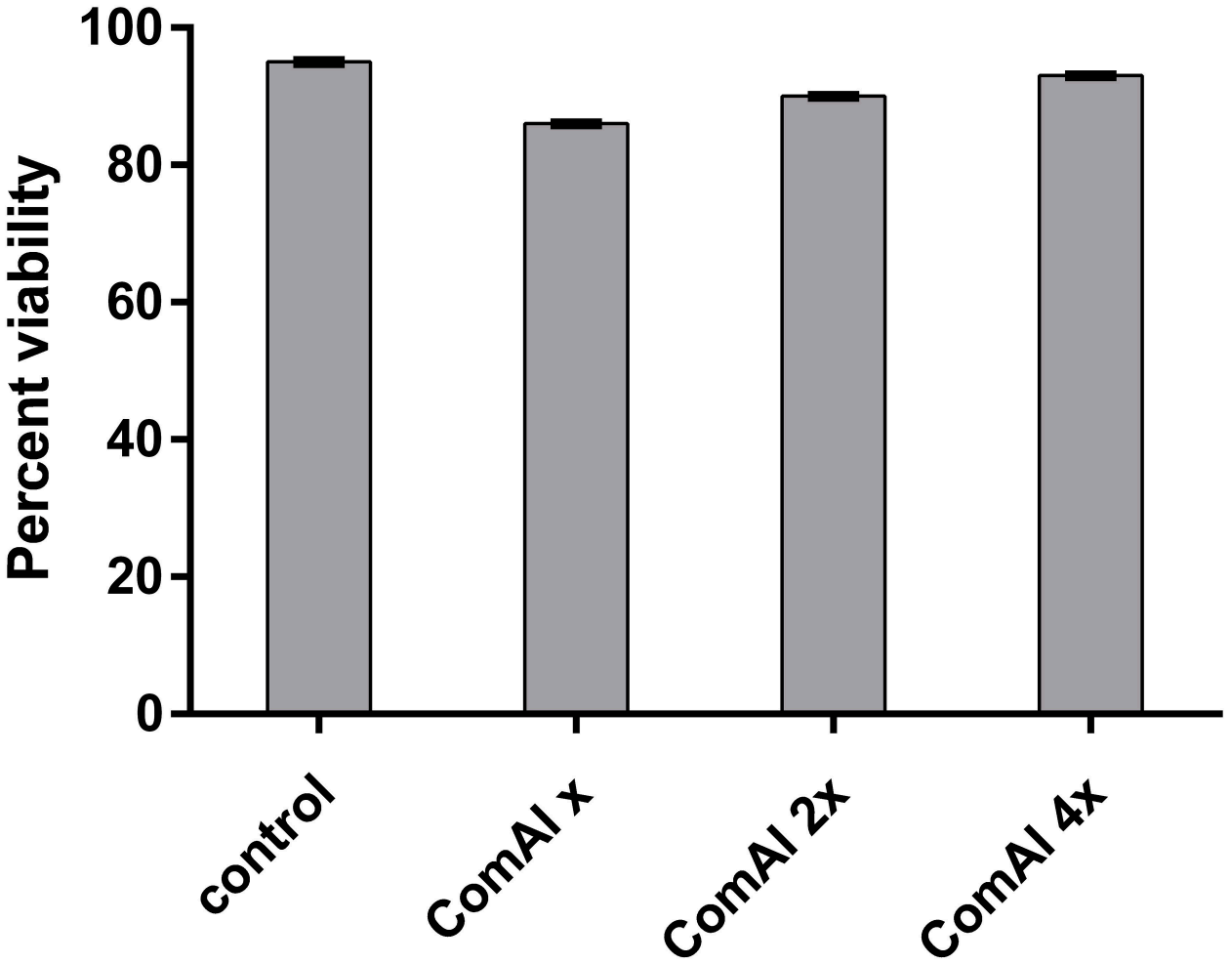
Viability of HepG2 cells after 24 h treatment. The cells did not exhibit any significant cytotoxicity with cells treated with DMTU at two as well as four times of the effective concentration (X = 3.75 μM).

In the present study, MTT assay revealed that DMTU and DMTU^1^ do not have any quantitative cytotoxic effect on morphology and proliferation of Hep G2 cell lines when compared with the respective control as shown in Figure 4. The cells were found to have about 90 percent confluence after 24 h of incubation. The results of cytotoxicity assay suggested that compounds can be further used in rodent animals for efficacy studies for validation of compounds.

### Acute oral toxicity studies

In acute oral toxicity study, the rats did not show toxic signs or death during the 14 day observation period. External examination of the rats did not show any signs of disease development and uptake of feed was normal without significant differences in the average weight gains among the experimental groups (data not shown). The skin and natural orifices of all experimental animals revealed no morphologic alterations. The animals did not show any variation in their general physical appearance and behavior and also, no signs of anorexia, depression, lethargy, jaundice, dermatitis throughout the study. Macroscopic observation of organs such as heart, lung, pancreas, spleen, liver, stomach, intestine, kidney, ovary, brain, eyes, and tongue revealed indifference among all the rats without any detectable pathological symptoms.

### DMTU reduce the incidence of dental caries *In vivo*

**Figure 5:**
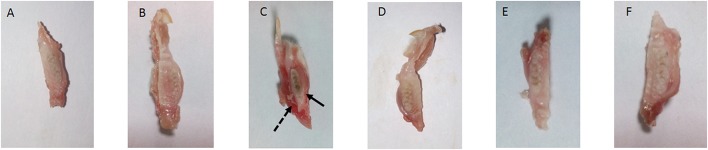
Incidence of dental caries. The image represents the occurrence of dental caries in various groups. **(A)** Normal control group without sucrose diet; **(B)** Normal control with sucrose diet; **(C)** Disease control group, solid arrow represents the occurrence of black lesions on molar crown indicating development of caries and dotted arrow represents the inflamed gum tissue due to infection by *S. mutans*; **(D)** DMTU (3.75 μM) treated group; **(E)** Fluoride (250 ppm) treated group; **(F)** DMTU (3.75 μM) along with fluoride (31.25 ppm) treated group.

**Figure 6:**
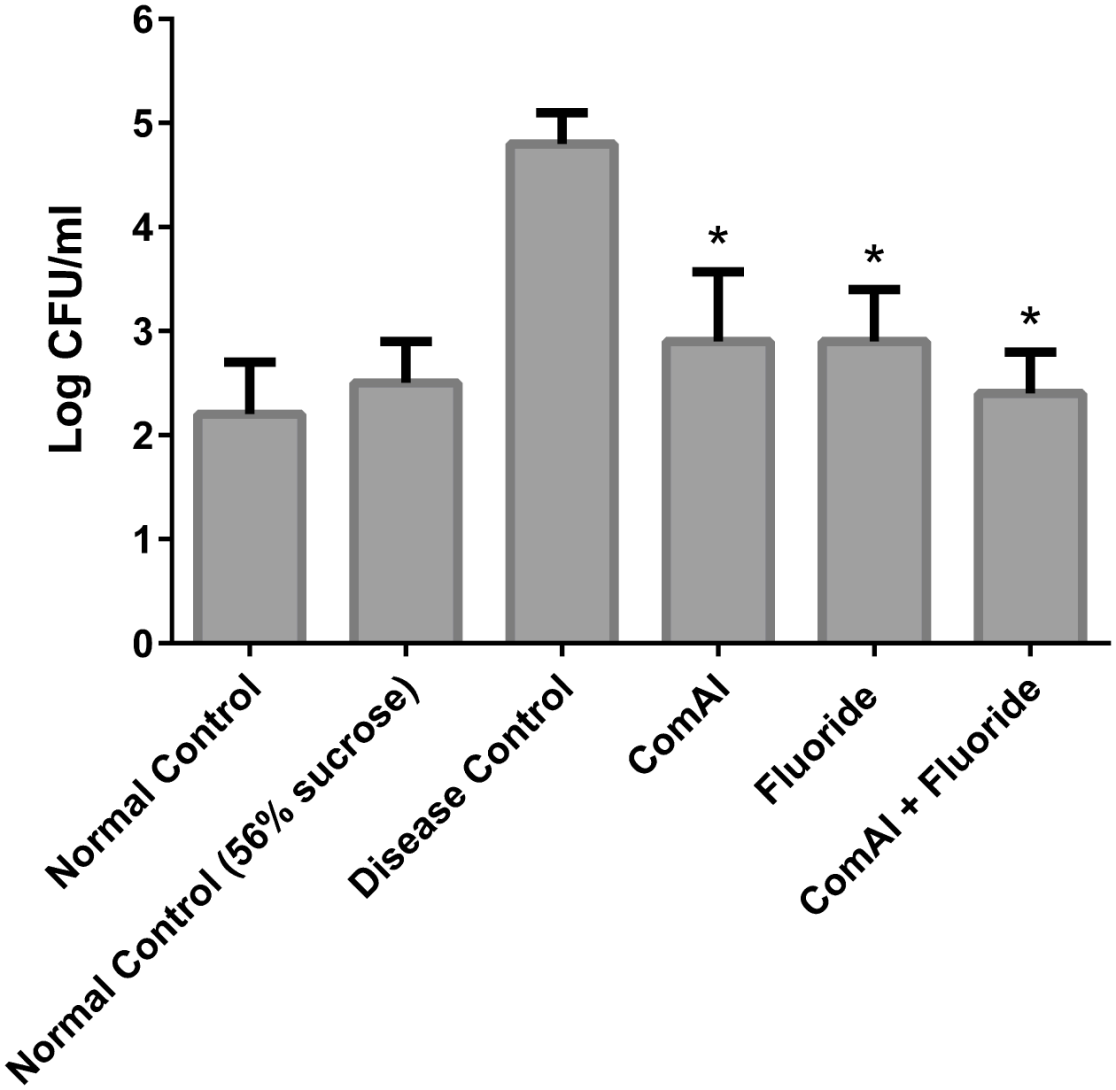
Anti-adherent activity of DMTU in treated groups in comparison to control groups was evaluated by colony forming unit (CFU) assay and the results were plotted on a logarithmic scale. The columns in the graph represent the mean of six animals per group. Error bars represent standard deviations with ^*^p < 0.05.

The present study has revealed that DMTU acts as a potential cariostatic agent and thus hinder the occurrence of dental caries *in vivo*. Macroscopic observations showed the development of brown and black lesions on the crown of diseased rats whereas, reduction in the development of lesions was found in DMTU treated group (Figure 5). Moreover, in the diseased group, the tissue around the molar root was inflamed as compared to the normal control group. The group treated with fluoride (250 ppm F, clinically proven anticaries agent) alone showed comparatively reduced lesions but not as significant as that of DMTU treated group. In this study, it is shown that DMTU (3.75 μM) in combination with lower concentrations of fluoride (31.25 ppm F) was considerably effective in reducing the occurrence of lesions and adherence of biofilm producing cells as compared to the fluoride alone. The colony count of SM4 showed significant reduction in the adherent cells in case of DMTU and combinatorial study group when compared with disease control (Figure 6). The total viable counts and the SM4 viable counts recovered from the rodents' plaque were not significantly affected by treatments with DMTU when compared to the normal control (*p* > 0.05).

### Histopathology studies

**Figure 7:**
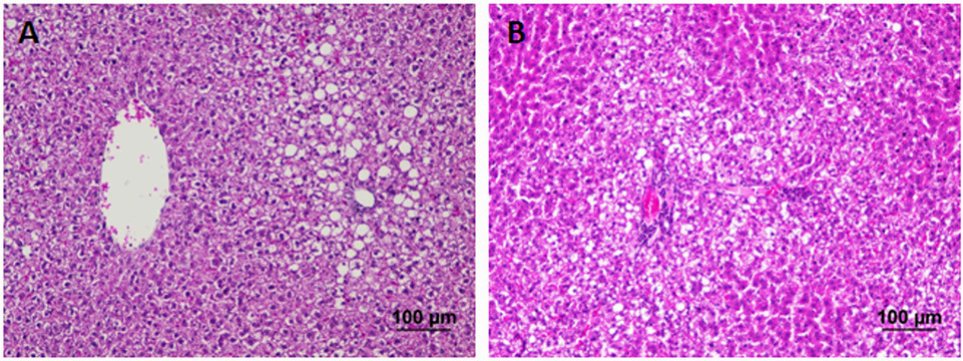
Hematoxylin and eosin staining of liver tissue: Hematoxylin stains the nucleus blue in color and counter staining by eosin imparts pink color to the cytoplasm. **(A)** Normal control group and **(B)** the group treated with 10 X dose of DMTU (X= 3.75 μM). Normal as well as treated groups showed normal liver tissue histology without any pathological signs (Section thickness: 3 μM).

**Figure 8:**
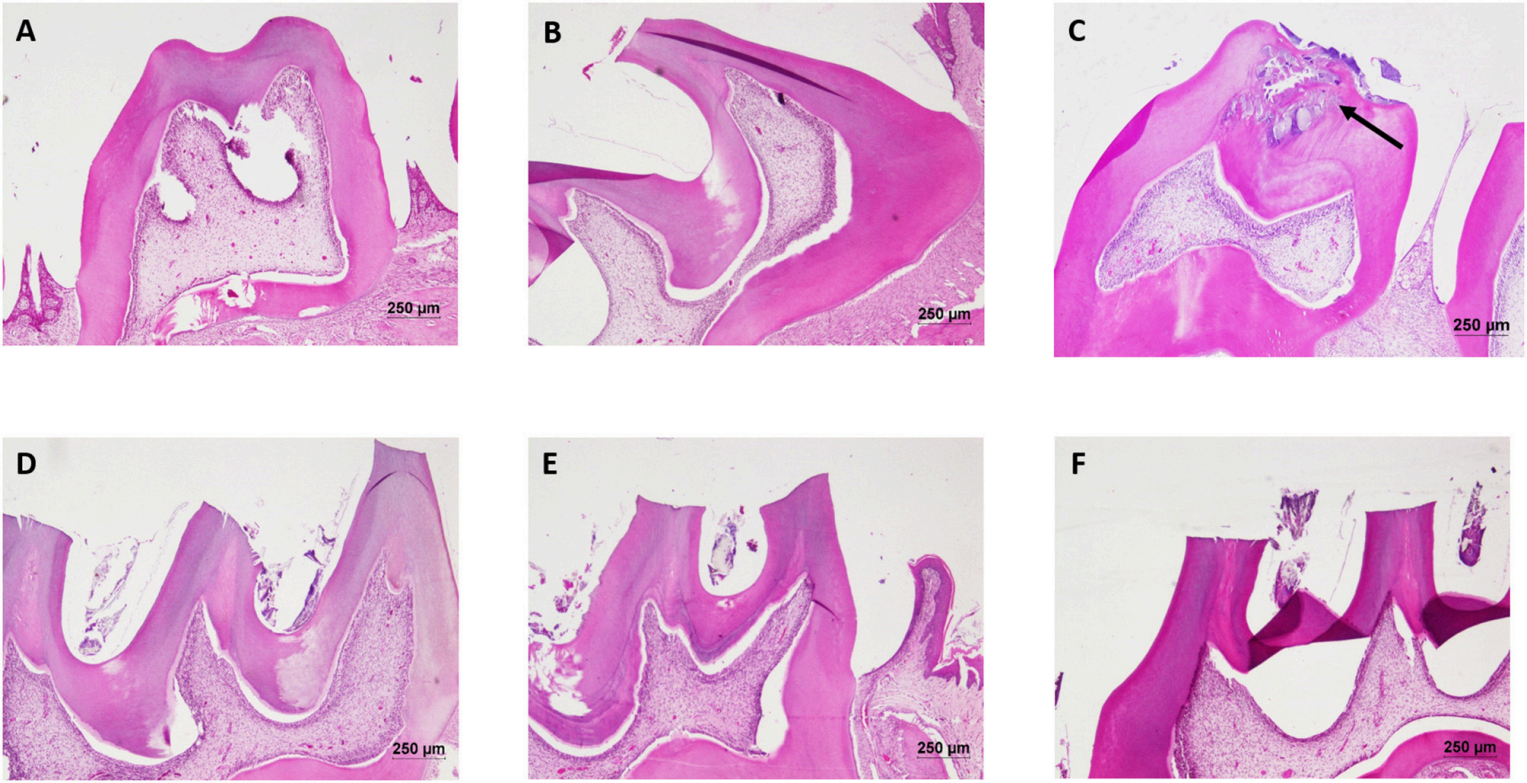
Hematoxylin and eosin staining of dental tissue: Hematoxylin stains the nucleus blue in color and counter staining by eosin imparts pink color to the cytoplasm. **(A)** Normal control group; **(B)** Normal control with sucrose; **(C)** Diseased group, solid arrow represents the lesions developed on the dentine and penetrated up to the dental pulp tissue; **(D)** DMTU (3.75 μM) treated group; **(E)** Fluoride (250 ppm) treated group; **(F)** DMTU (3.75 μM) along with fluoride (31.25 ppm) treated group (Section thickness: 3 μM).

The liver of control as well as DMTU (10 X dose; X = 3.75 μM) administered rats showed normal hepatic structure, characterized by polygonal-shape hepatocytes with well-defined boundaries, large centrally located nucleus with light stained acidophilic cytoplasm along with dispersed chromatin radially disposed in hepatic lobules (Figure 7). The incidence of dental lesions is summarized in Figure 8. Decalcified longitudinal sections of teeth of the normal group showed healthy dentine, odontoblast, and pulp, whereas in the diseased group, the carious dentine lesions were moderate to severe transcending through odontoblast into the pulp and completely decayed enamel crown. Almost no carious lesions were detected in DMTU treated as well-combinatorial treated group whereas moderate carious lesions were recorded in fluoride treated group.

### Reduction in inflammatory markers

**Figure 9:**
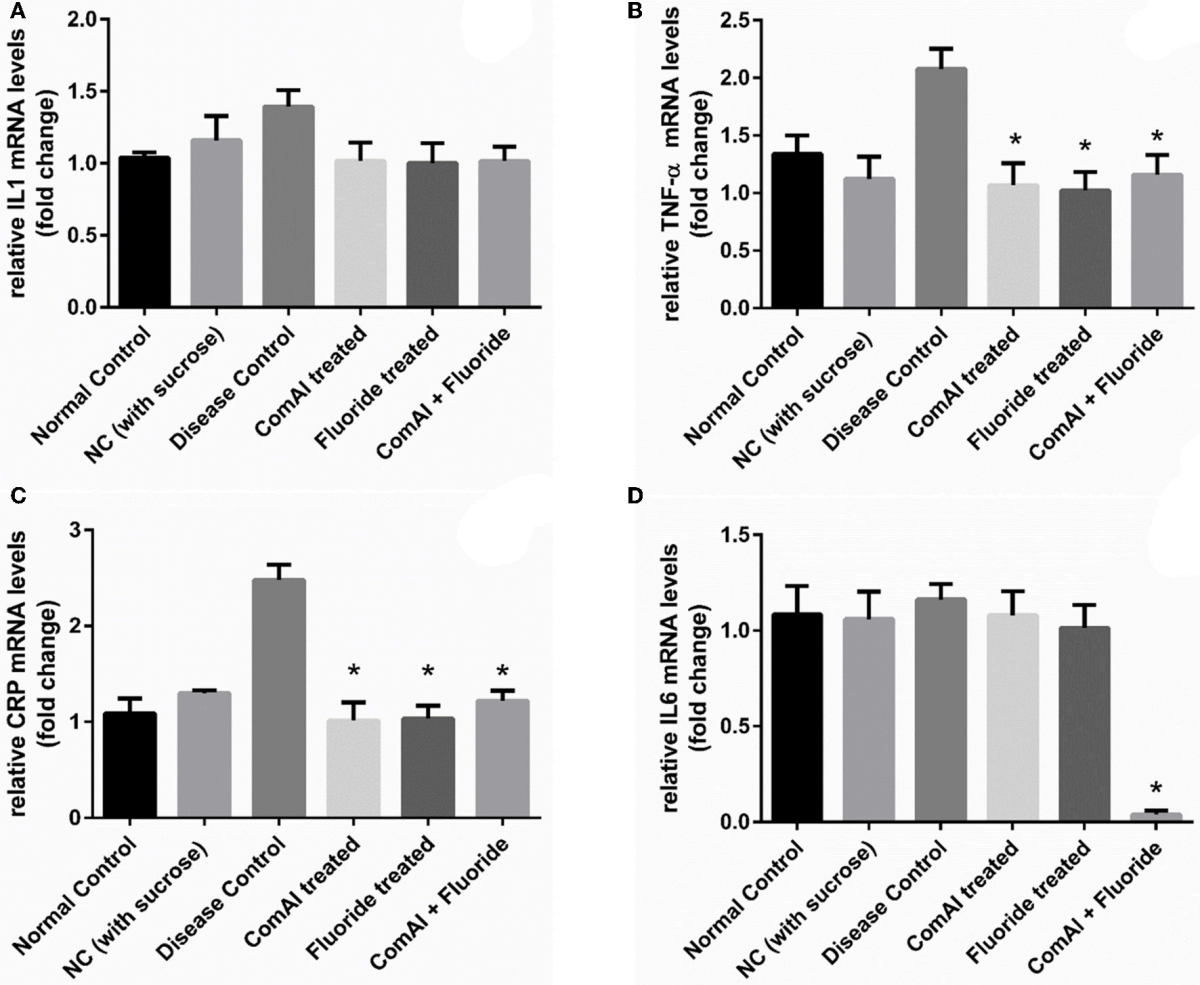
RT-qPCR analysis of inflammatory markers in liver tissues of Wistar rats. **(A)** IL-1; **(B)** TNF-α; **(C)** C-Reactive Protein (CRP); **(D)** IL-6. The results are represented as ratios corresponding to the fold change in various treatment groups when compared with normal control group (without treatment). *Gapdh* gene was used as an internal control for data normalization. ^*^Indicates significantly different (*p* < 0.05) compared to untreated tissues.

**Figure 10:**
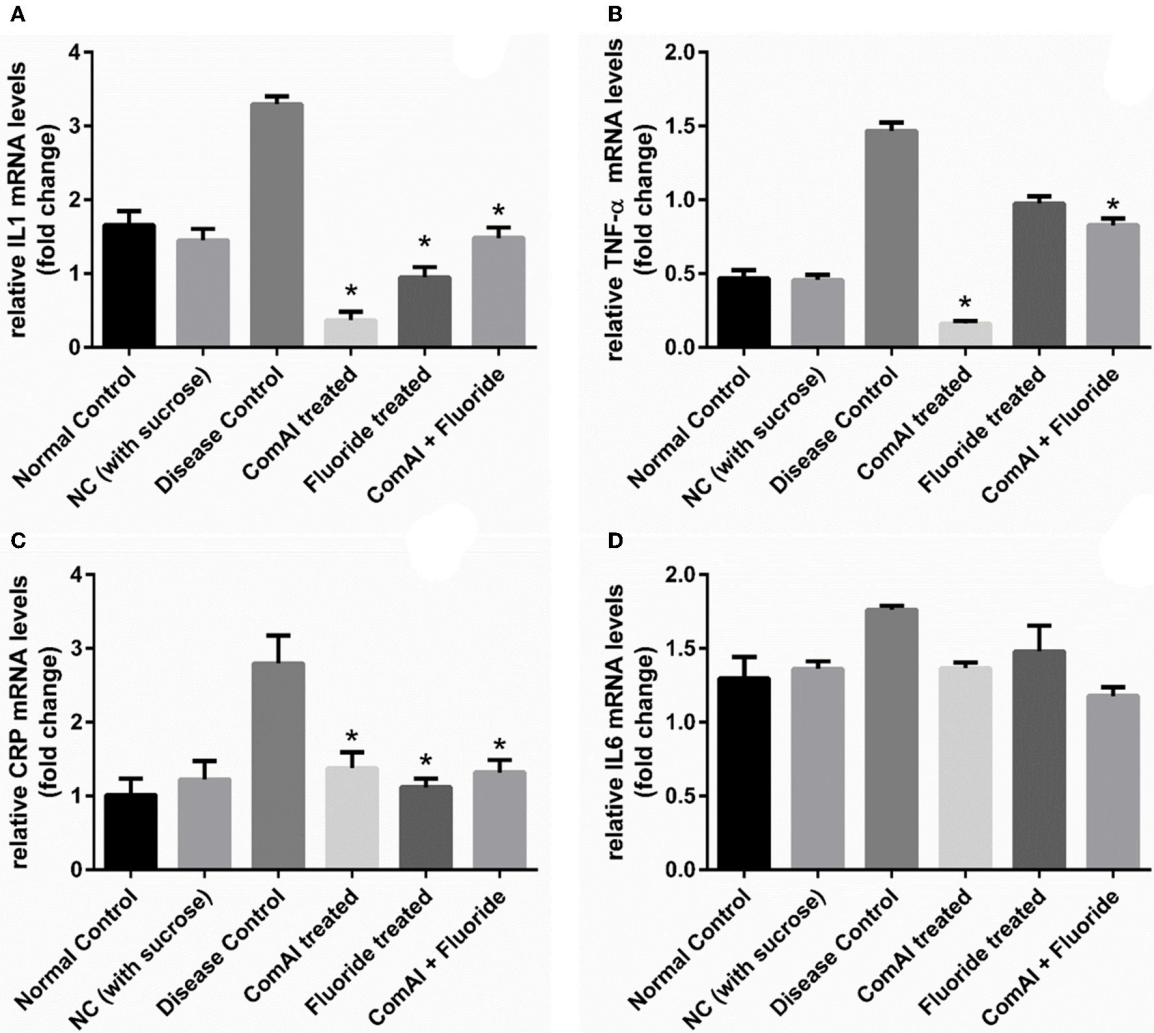
qRT-PCR analysis of inflammatory markers in samples collected from the blood plasma of Wistar rats. **(A)** IL-1; **(B)** TNF-α; **(C)** C-Reactive Protein (CRP); **(D)** IL-6. The results are represented as ratios corresponding to the fold change in various treatment groups when compared with normal control group (without treatment). *Gapdh* gene was used as an internal control for data normalization. ^*^Indicates significantly different (*p* < 0.05) compared to untreated tissues.

Inflammatory markers such as IL-1, IL-6, TNF-α, CRP showed varying expression levels in diseased as well as treated groups. In case of liver samples (Figure 9), levels of proinflammatory cytokines TNF-α, CRP, IL-1, and IL-6 were found significantly elevated in diseased group as compared to the control group (*p* < 0.05). Treatment with DMTU and DMTU along with fluoride significantly (*p* < 0.05) decreased the expression of TNF-α, CRP, IL-1. However, IL-6 levels were not affected by DMTU treatments but DMTU along with fluoride was able to reduce the expression significantly. Furthermore, in plasma (Figure 10), except IL-6, other inflammatory markers used in this study, i.e., IL-1, CRP, and TNF-α showed significant reduction in expression levels in treated groups (DMTU alone and Combinatorial group) as compared to the diseased group.

## Discussion

Oral cavity is one amongst the dynamic microbial community niche consisting of more than 700 species in equilibrium. Most of the species are commensal and help in maintaining the normal balance and thus avoiding pathogenic interference by opportunistic pathogens. Emergence of multidrug resistant strains has raised the concern and need for the development of better anti-virulent drugs. In this context, our present study focussed on the *in vitro* and *in vivo* validation of target specific anti-virulent drugs which were previously reported by our research group to have better binding to DMTU (Kaur et al., 2016).

Our study examined the effects of DMTU and fluoride at mid-logarithmic growth phase and stationary growth phase. The genes considered in this study are reported to be directly and indirectly involved in quorum sensing circuit of *S. mutans*. At mid-log phase, the expression of *immA* and *immB* (bacteriocin-immunity genes) were found to be up-regulated. Similar results were observed previously by Wang et al. (2013) where the group reported up-regulation of *immA* and *immB* genes upon treatment with chlorhexidine in *comC* mutant. Additionally, they also reported the enhanced sensitivity of *comC* mutant toward antimicrobials indicating the indirect involvement of quorum sensing in resistance toward various antimicrobials. In a previous study by Sztajer et al. (2008) the effect of the *luxS* mutant on the expression of bacteriocin genes was explored and was in-line context with our present data except for the *bsmH* gene which was found to be up-regulated in their study. This can be attributed to the fact *luxS* might be regulating the expression of *bsmH* in an alternative way and not through the two component ComDE TCTS system. Parallel reports by Banu et al. (2010) showed the down-regulation of *bsmH* as well as other bacteriocin related genes in *pknB* mutant strains. They have speculated that *pknB* modulates the activity of ComDE TCTS system. On the other hand, as expected, treatment of MTCC 497 and SM4 strains with DMTU, resulted in down-regulation of the genes involved in competence development and bacteriocin production through ComDE quorum sensing pathway. The effect of DMTU further transcended till stationary phase indicating that the effect is not temporary and has effect at later stages of growth in *S. mutans*. The *comA* gene was found to be down-regulated as *S. mutans* enters from early to mid and then stationary phase as a result of positive feedback loop present in ComDE QS pathway. Interestingly, on exposure to fluoride alone, the relative expression of the genes was found to be at the basal level when compared with the control samples. This signifies that fluoride does not have any effect on the ComDE pathway and it might be affecting alternative pathway involved in the EPS production as well as sucrose metabolism.

MTT assay was carried out to evaluate the potential toxicity of DMTU in cell lines before proceeding for *in vivo* acute oral toxicity in Wistar rats. The *in vitro* cytotoxic results revealed that DMTU was not found to have any toxic effect on mammalian cell lines making it suitable for validating the drug *in vivo*. In acute oral toxicity studies, the rats were found to be healthy till the highest dose used (2,000 mg/kg/PO) as per OECD 425 guidelines. This shows that DMTU does not have short-term as well as long-term toxic effects.

The efficacy study was carried out to evaluate whether the antibiofilm and cariostatic activity of DMTU would be similar to that of *in vitro* study, with widely used Wistar rat model for dental caries study *in vivo* (Koo et al., 2005). Topical application of DMTU significantly reduced the formation of biofilm and effectively decreased the incidence of dental caries when compared with disease control (*p* < 0.05) confirming previous *in vitro* findings (Kaur et al., 2016). The property of DMTU in reducing the development of carious lesions on the tooth surface clearly indicates anti-caries activity at the brief exposure of efficacious concentration in the presence of sucrose-rich diet when ingested by the animals when compared with disease control (*p* < 0.05). The ability of topically applied DMTU to have a persistent anti-caries effect is a desirable characteristic of a novel chemotherapeutic agent targeting biofilm oriented dental diseases such as dental caries (Bowen, 2015). It is noteworthy that colony counts of the total microflora of the oral cavity and the SM4 *S. mutans* were not affected which approves well with its lack of antibacterial activity against biofilm results of our previous findings (Kaur et al., 2016). Furthermore, these observations indicate that the caries preventive mechanisms of DMTU may be related to its effects on ComA in quorum sensing circuit resulting in down-regulation of several virulence attributes of *S. mutans*, such as biofilm formation and bacteriocin production by this pathogen. Combinatorial study group in rodent model showed that DMTU in combination with fluoride enhances the anti-cariogenic effect of fluoride thus, clearly has potential clinical application to reduce the prevalence of dental caries at lower concentrations without increasing the concentration of fluoride exposure. As mentioned earlier, fluoride does not alter the expression of genes involved in quorum sensing of *S. mutans*. In previous reports, fluoride levels found in plaque, affect the glycolytic activity and production of Gtfs by disrupting the proton permeability of the cell membrane in *S. mutans* (Marquis et al., 2003; Koo et al., 2006). The intracellular polysaccharides (IPS) is metabolized by oral pathogens when external sources of fermentable carbon have been depleted in the oral cavity. Thus, IPS promote the occurrence of carious lesions by enhanced exposure of tooth surfaces to lower pH in the biofilm niche. IPS is synthesized as a result of ATP pools in cells of the biofilm matrix. Fluoride significantly reduces the ATP pools and results in substantial reduction in IPS synthesis and as a result reduces the incidence of lower pH in the oral cavity. In addition, fluoride also enhances the remineralization process and cause a reduction in the demineralization process at the tooth-biofilm interface. The present data not only corroborate previous *in vitro* findings but also support the hypothesis that interfering with the quorum sensing circuit possibly results in a reduction of caries by inhibition of virulence attributes and biofilm formation by *S. mutans*.

The liver is one of the major organs involved in the detoxification of the body. It was necessary to evaluate the long-term effects of higher doses of DMTU on hepatocytes to eliminate any toxicity pattern involved with the administration of DMTU. In the present study, liver sections did not show any degenerative signs thus, proving the administration of DMTU (up to 10 X doses for long term might not be toxic to the recipients at clinical stages. Histopathological examination of teeth and absence of lesions in DMTU treated group provides an evidence that topical applications of DMTU and in combination with fluoride have a cariostatic effect. In disease group, caries has penetrated enamel (decalcified), dentine and reached the depth of teeth i.e., pulp (soft connective tissue; Figure 8). The entrance of *S. mutans* in pulp can lead to the invasion of bacteria in the blood stream causing a systemic pro-inflammatory response in the body (Nakano et al., 2004). A review study by Esser et al. (2014), has reported the potential link between low-grade chronic inflammation and systemic diseases such as obesity and Type 2 Diabetes. High-level expression of the inflammatory response may lead to the development of systemic diseases such as cardiovascular disease, rheumatoid arthritis, type 2 Diabetes, premature birth of babies and so on (Gurenlian, 2009). To investigate this, we estimated the expression levels of inflammatory markers such as TNF-α, IL-6, IL-1, CRP in plasma as well as liver tissues. The liver has a remarkable capacity to adapt to injury stress through tissue repair when compared to any other solid organ in the body. Complex interactions of immune cell subsets regulate this repair process. Levels of pro-inflammatory cytokines TNF-α, CRP, IL-1 were significantly down-regulated in the case of plasma as compared to liver, attributing to the fact that, cytokines are more readily diffused and easily estimated by RT-qPCR analysis as compared to liver tissues. The low levels of inflammatory markers in case of treated groups can be attributed to the fact that due to inhibition of biofilm formation by DMTU alone and in combination will prevent the formation of carious lesions and further inhibit the invasion of *S. mutans* in the pulp.

Collectively, the data in our present study shows that DMTU reduces the incidence of caries development by targeting the ComA in quorum sensing pathway of *S. mutans* which further affects the major virulence factors such as biofilm formation, bacteriocin production without causing mortality of bacteria. DMTU along with lower concentrations of fluoride could be used as a potential cariostatic measure to reduce the incidence of caries without affecting the remineralization property of fluoride. The combination of DMTU with fluoride at lower concentrations may provide a potential substitute to the current chemotherapeutic approaches to prevent the incidence of dental caries. In addition, prevention of caries also results in the reduction of inflammatory markers as shown in this study at pre-clinical stages. Additional studies are warranted to link and explore pathways that link dental caries to systemic diseases and this may provide a guide to further enhance the anti-inflammatory chemotherapeutic anti-caries agents in oral formulations.

## Author contributions

All authors listed have made a substantial, direct and intellectual contribution to the work, and approved it for publication.

### Conflict of interest statement

The authors declare that the research was conducted in the absence of any commercial or financial relationships that could be construed as a potential conflict of interest.
